# The Impact of Sebum and Pore Size on Consumer Perceptions of Skin Yellowness Among Young Chinese Consumers

**DOI:** 10.1111/jocd.70709

**Published:** 2026-02-02

**Authors:** Sharon Shi, Linda Ruan, Bee Leng Lua

**Affiliations:** ^1^ Groupe Clarins Singapore Singapore

**Keywords:** claim substantiation, dull skin tone, pores, sebum, skin physiology, skin yellowness perception

## Abstract

**Background:**

Perceived skin yellowness is a common skin concern among consumers in China. Existing cosmetic solutions focus on whitening and anti‐aging, while the causes of perceived skin yellowness in young consumers are unclear. This study investigated how sebum amount and pore size influence the perception of skin yellowness in young Chinese consumers.

**Methods:**

This study had two phases. Phase 1 involved capturing photographs and measuring skin parameters every 2 h over a 12‐h period in Chinese women aged 18–35 years with dry (*n* = 31) or oily skin (*n* = 33). For Phase 2, representative images of four Phase 1 oily skin participants (two fair‐ and two dark‐skinned) were modified using artificial intelligence (AI) gradient processing based on three test parameters: pore size, sebum amount, and pore size + sebum amount. Respondents (*n* = 200) assessed the test parameters' influence on skin yellowness using these images.

**Results:**

In Phase 1, individual typology angle (ITA°) decreased as sebum production increased over the 12‐h for both skin groups, with a stronger correlation in the oily skin group. In Phase 2, perceptions of skin yellowness increased with increasing sebum amount and pore size + sebum amount. This result was more prominent with dark skin tones. Pore size alone modestly affected perceived skin yellowness.

**Conclusions:**

Sebum and, to a lesser extent, pore size increased the skin yellowness perception among young Chinese consumers. These findings enhance the understanding of skin yellowness in younger people and will aid in developing future cosmetic solutions for these concerns.

## 1 Background

Societal conventions for facial skin tone and complexion significantly influence the perception of attractiveness and age in female faces [1]. In addition to its biological and protective function, healthy appearing skin is associated with well‐being and good social integration [2]. In East Asia, an even, fair, and glossy skin tone is often perceived as a sign of beauty, health, and youth [1], while skin yellowness is perceived as undesirable and is commonly associated with aging and a dull skin complexion [3]. Aside from aging, factors such as genetics, lifestyle, UV radiation exposure, climate, and skin biophysical parameters including sebum, pores, and hydration have an impact on skin tone [4]. While skin anti‐aging and whitening efficacy products and routines already exist for Chinese consumers concerned with skin yellowness, there is a need for cosmetic products that specifically target perceived skin yellowness among younger Chinese consumers.

One feature that has been associated with skin yellowness is excess sebum production (i.e., oily skin), which is also a common skin concern among young consumers [5]. Excessive sebum, or oily skin, is considered an undesirable skin type and is often associated with increases in pore size, acne, excessive shininess, and lackluster skin pigmentation, in particular skin yellowness [2, 6, 7]. Sebum oxidizes relatively easily, especially when exposed to sunlight, and may cause skin dullness in a fairly short time frame [7, 8]. The rate of sebum production is highest between puberty and age 35 and steadily declines from about 40 years of age [9, 10]. Sebum production can vary significantly across the facial topography. Sebum production is highest in the T‐zonal area of the face (forehead, nose, and chin) and lowest in the U‐zone (right cheek to left cheek) [5].

Pores are another feature associated with skin yellowness. A pore is a microphotographic feature on the skin surface that corresponds to an enlarged pilosebaceous unit opening [2]. Pores release the sebum secreted by the sebaceous glands [11]. The pore size is determined by both intrinsic and extrinsic factors; however, enlarged facial pores are a cosmetic concern for many consumers [4]. The effect of pores on skin yellowness has been linked to sebum levels; when sebum production is excessive, pores can become blocked, and this can cause them to appear larger or deformed [11]. The presence of enlarged pores can be positively correlated to the amount of sebum produced [2, 4, 12]. Furthermore, the outer layer of the epidermis of a pore is continually in contact with sebum [13]. As a result of this contact, corneocytes around the pores can accumulate carbonylated proteins from sebum, which may darken the appearance of the pores and ultimately dull skin appearance [13]. Enlarged pores can also cause an uneven skin surface, and this can create differences in the reflective index, leading to the appearance of dull or yellow skin [14, 15, 16]. Moreover, a larger pore area and greater skin roughness are associated with an increased perception of skin dullness [17]. This correlation between skin texture and dullness is more pronounced in younger women [17].

The effects of excess sebum and pores on the appearance of skin tone are thought to be rapid; significant changes in sebum secretion and skin pores have been shown to occur within just 4 h, and this increase is more marked in people with oily skin compared to those with dry skin [16]. These increases in sebum and pores were shown to significantly increase skin redness and roughness in people with oily skin, but not in people with dry skin [16], suggesting that people with oily skin are more greatly impacted by excess sebum and pores.

Evidence suggests that sebum and pores influence facial skin yellowness based on skin physiology; however, it is unclear whether these changes can be perceived by consumers. It is also unclear which of these parameters have the greatest influence on consumer perception of skin yellowness. This study focused on the impact of sebum and pores on perceived skin tone. This study was divided into two phases. In Phase 1, we captured short‐term (≤ 12 h) facial sebum accumulation in young Chinese women and assessed whether skin tone and yellowness were altered over time. Based on the results from Phase 1, in Phase 2, we assessed how young Chinese women visually perceive skin yellowness as sebum amount, pore size, and pores size + sebum amount increase. This study focused on young women (aged 18–35 years) since the rate of sebum production is highest in adults under 35 years of age [9, 10]. The findings of this study will support the development of cosmetic products to address skin yellowness concern among younger people.

## 2 Methods

### 2.1 Study Overview

This was an observational study that investigated the subjective assessment of skin yellowness in relation to pore size and sebum amount in healthy Chinese women between November and December 2024. The study was conducted in Shanghai and involved two phases: clinical measurements and photo taking (Phase 1) and consumer evaluation (Phase 2). All participants provided their informed consent before participating in the study.

### 2.2 Phase 1

Phase 1 was conducted to gain a better understanding of how different skin parameters affect skin yellowness in participants with either dry or oily skin in the short‐term. A 12‐h test period was designed to mimic and reflect consumers' real skin concerns in their daily life by capturing the maximum facial sebum accumulation within 1 day.

### 2.3 Phase 1—Participants

Sixty‐four participants completed the study and were divided according to their skin type into either the oily skin group (*n* = 33) or dry skin group (*n* = 31). Within each group, the participants were further sub‐divided by age (18–25 years old and 26–35 years old). Inclusion and exclusion criteria are summarized in Table 1.

**TABLE 1:** Participant inclusion and exclusion criteria for Phase 1 and Phase 2.

Phase 1‐inclusion criteria		
Age	Aged 18–35 years	
Gender	Female	
Skin type	Dry or oily skin	
ITA^o^	> 28	
**Phase 1—exclusion criteria**		
Pregnant or breast‐feeding women Taken anti‐inflammatory medication within 2 months Taken any medication or treatment that affect sebum secretion within past 3 months Inflammatory skin that has not clinically recovered Received hormone therapy that affected sebum production in the past 6 months Self‐declared sensitive and redness‐prone facial skin		
**Phase 1—baseline characteristics**		
	Dry skin group	Oily skin group
Sample size (n)	31	33
Age (years, mean)	26.87	26.70
Sebum value (μg/cm^2^, mean)	20.42	84.67
SC hydration (Corneometer)	36.25	N/A
**Phase 2—inclusion criteria**		
Age	Aged 18–35 years	
Gender	Female	
Skin type	All skin types Self‐declared yellowness concerns Minimum of *n* = 60, self‐declared dry skin (dry skin or normal‐to‐dry skin) Minimum of *n* = 60, self‐declared oily skin (oily skin or normal‐to‐oily skin)	
Education level	High school and above	
Ability to discriminate color	Must pass	
**Phase 2 – exclusion criteria**		
Noncompliance with consumer research industry practices Taken part in consumer research in the past 3 months Pregnant or trying to get pregnant Individuals who are nursing		

Abbreviations: ITA°, individual typology angle; SC, stratum corneum.

### 2.4 Phase 1—Data Collection for Screening

During consumer recruitment, skin sebum value and stratum corneum (SC) hydration value were collected to determine participant skin type for skin type allocation. A sebum value from a participant's cheek (randomized) was obtained 1 h after facial cleansing. SC hydration values were obtained from an average of 3 measurements per cheek (left/right) using a Corneometer CM825 (Courage and Khazake, Germany). Participants with an SC hydration value of < 52 Corneometer units and an initial sebum value of < 40 μg/cm^2^ were allocated to the dry skin group. Participants with a sebum value of > 40 μg/cm^2^ were included in the oily skin group [5, 18]. To ensure each group had the same average skin tone, skin tone and yellowness were measured as individual typology angle (ITA^°^), using a spectrophotometer (CM‐26dG, Konica Minolta (China) Investment Ltd.) on the participant's right cheekbone area. ITA^°^ is an objective measure of skin pigmentation. ITA^°^ has a strong inverse correlation with melanin index, whereby skin with higher melanin concentrations (i.e., high pigmentation) exhibits a lower ITA^°^ [19, 20]. Each participant's skin was also assessed by Fitzpatrick type to ensure each group had a similar range of Fitzpatrick type distribution (Type 1 ITA^°^ > 55^°^, Type 2 ITA^°^ > 41^°^ to 55^°^, Type 3 ITA^°^ > 28^°^ to 41^°^). Lastly, participants in the oily skin group had sebaceous pores on their cheek area assessed by a dermatologist using SKIN AGING ATLAS volume 2 Asian Type [21]. They were then grouped and divided into smaller pore (≤ GRADE 2) and larger pores (≥ Grade 3) to ensure both smaller and larger pore sizes were included in the oily skin group.

### 2.5 Phase 1—Data Collection for Formal Testing

Participants underwent facial cleansing at the beginning of the test period. Clinical measurements (ITA^°^, sebum, and hydration) and photographs of participants' faces were collected every 2 h over a 12 h test period (0 h, 2 h, 4 h, 6 h, 8 h, 10 h, and 12 h). Skin sebum values were obtained from a participant's central forehead area, and ITA^°^ and SC hydration were measured as previously described. Deep hydration values were obtained from the average of 3 measurements per cheek using a MoistureMeterD with an S15 probe (Delfin, Finland). Facial images were captured every 2 h using VISIA‐CR (Canfield, American). The images and the skin classification data were used to generate images for the Phase 2 visual perception study.

### 2.6 Phase 2‐ Visual Perception

#### 2.6.1 Phase 2—Development of Test Images

Based on the results of Phase 1 and the demonstrated association of sebum and skin yellowness in oily skin, sebum amount was selected as a parameter for Phase 2. Pore size was also chosen due to the association of pore size to skin yellowness and sebum amount, as discussed in the literature [2, 4, 11, 12, 13]. To assess the impact of pore size, sebum amount, and the combination of pore size + sebum amount on the perception of skin yellowness, images were generated with the AI technique of texture blending (Programming Language: Python 3.9.5., Main Libraries: Open CV(4.5.2.), NumPy(1.20.1.)) [22, 23]. For the development of AI‐generated images for Phase 2, the 12 h post‐cleansing photos of four participants from the Phase 1 oily skin group were used as the baseline. The technique of texture blending was used to create six photos, with each image differing in the gradient of one influencing parameter: pore size, sebum amount, or pore size + sebum amount. Pores were evenly reduced from baseline (100%) by 20% per level until no visible pores (0%). Similarly, sebum levels were evenly reduced from baseline per level until initial post‐cleansing status.

The reason oily skin group models were exclusively chosen was because the Phase 1 results indicated that sebum was the primary driver of skin tone change for oily skin in the short term (i.e., over 12 h). Four varying dimensions (different skin tone, age group, sebum amount, and pore size) were factored into model selection to minimize test bias. As such, the four models chosen (Suppl. Figure 1) included two models with Type 2 skin (fair skin) and two models with Type 3 skin (darker skin).

### 2.7 Phase 2—Participants

In total, 200 participants were screened with the inclusion criteria (Table 1) for Phase 2. The participants were aged between 18 and 35 years and were divided into two age groups: 18 to 25 years old (*N* = 100) and 26 to 35 years old (N = 100). Participants included in the Phase 2 study were asked to self‐report their own skin conditions including skin type, pore size from type 1–6 (using reference images from SKIN AGING ATLAS volume 2 Asian Type), Fitzpatrick skin type (using provided skin type descriptions), and their skin concerns including skin yellowness (Suppl. Figure 2). The participants were also screened using two onsite tests. First, the participants were given color vision examination plates to screen for and exclude those who had difficulty distinguishing color. The participants were then tested on their ability to distinguish between different real human skin tones. This test included four images with varying skin tones that were artificially modified from images of a fair‐skinned model and a model with more yellow‐toned skin. The participants were asked to select the fairest and most yellow image from each model. A minimum pass rate of 50% for each model was required for inclusion in the formal testing phase.

### 2.8 Phase 2—Data Collection

**FIGURE 1:**
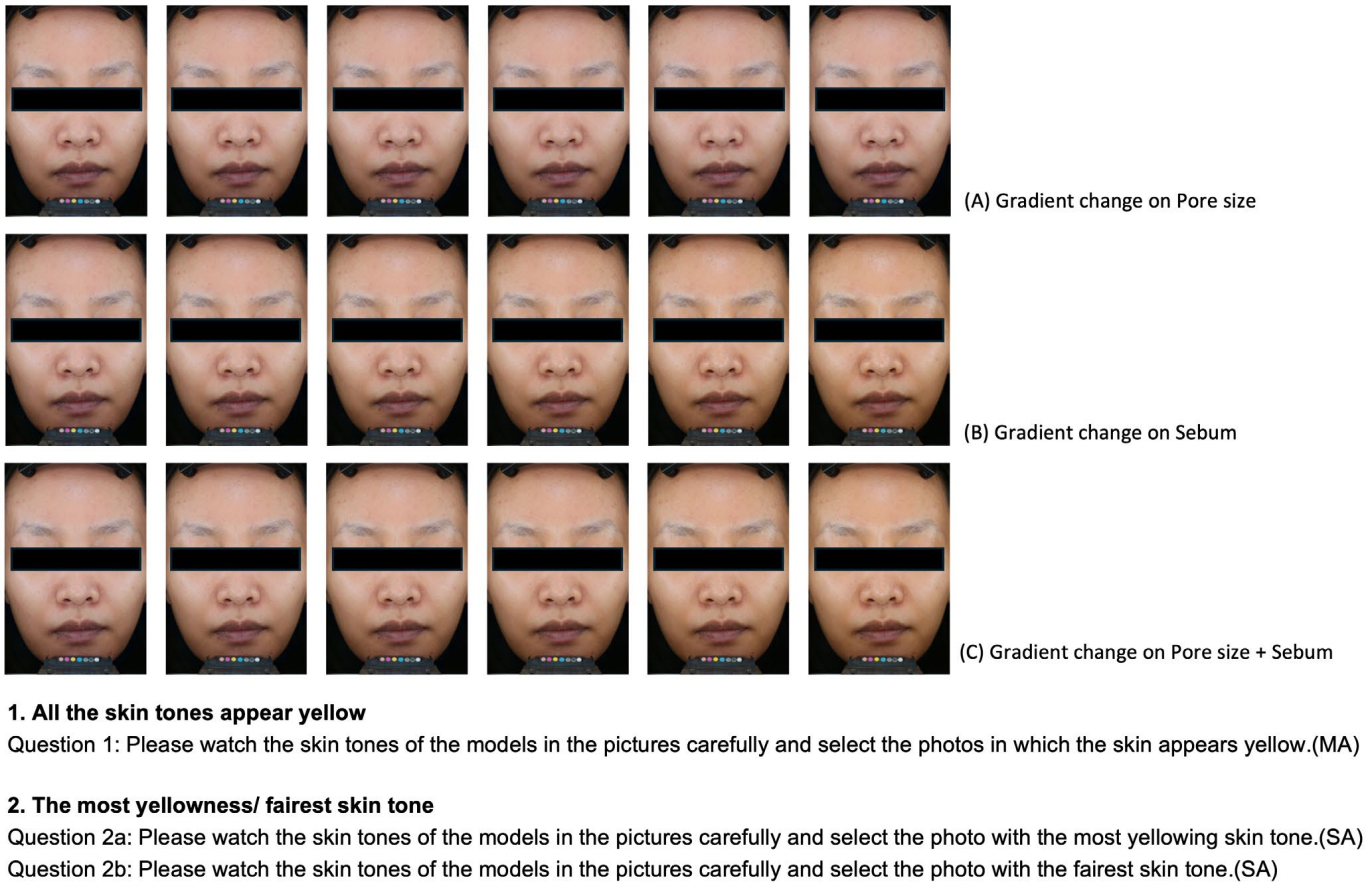
Visual perception Study‐Participant Questionnaire.

This phase was conducted to test consumer perceptions of skin yellowness with consideration to the three skin parameters: pore size, sebum, and pore size + sebum amount. The visual perception study had two arms (Figure 1): yellowness perception with a gradient change (multiple‐choice to select for images that appear yellow) and 2 dimensions for yellowness perception (single choice for most yellow and most fair). The multiple‐choice questions were used to demonstrate the distribution of overall consumer yellowness awareness across the gradients of each influencing factor, which reflected consumers' sensitivity to yellowness regarding the gradient change. For the multiple‐choice test, participants were shown a randomized series of six images of the same model, with each image differing in the gradient of one influencing parameter: pore size, sebum amount, or pore size + sebum amount. The participants were required to choose the photos in which their skin appeared yellow. There were four sessions (with 4 models) for each parameter, totaling 12 sessions for the multiple‐choice test. The single choice test was used to examine the prevalence of the consumers' ability to distinguish skin yellowness in relation to the specific parameters. For the single choice test, each participant was shown a series of six images of the same model, with each image differing in the gradient of an influencing parameter: pore size, sebum amount, or pore size + sebum amount. The participants were asked to select the image with the most yellow skin tone and the image with the fairest skin tone. In the single choice test, each session contained two questions (most yellow and most fair) for each set of photos; there were four sets of photos used per parameter, totaling three sessions with eight questions per session.

## 3 Analysis

### 3.1 Phase 1

In Phase 1, clinical data were analyzed by using Student's *t*‐test (normal distribution) or Wilcoxon if non‐normally distributed. The ITA^°^, skin sebum, SC hydration, and deep hydration values were used for the correlation analysis of both the dry and oily skin groups.

### 3.2 Phase 2

For the results of the multiple‐choice test, the scores of skin yellowness were assessed against the six gradients for each skin parameter and plotted separately for each parameter. Then, the results from each parameter were sub‐analyzed by skin tone of the model (fair versus dark) and plotted to compare the results of fair versus dark skin in perceived skin yellowness. To compare parameters, the yellowness scores for pore size, sebum amount, and pore size + sebum amount were plotted together. The data from the single choice test were plotted by most yellow skin tone and most fair skin tone for each parameter. The results of all parameters were then plotted together to compare to the results of most yellow skin tone and fairest skin tone. Data were analyzed in SPSS by performing a descriptive statistical analysis including mean, standard deviation, standard error, median, and minimum and maximum values. The analysis was performed using the Cochran's Q test to examine differences in proportions across related groups. Significance was determined by *p* ≤ 0.05 which corresponds to a 95% confidence interval.

## 4 Results

### 4.1 Phase 1 Study

#### 4.1.1 Factors Influencing Perceived Skin Yellowness in Young Chinese Consumers With Dry and Oily Skin

**FIGURE 2:**
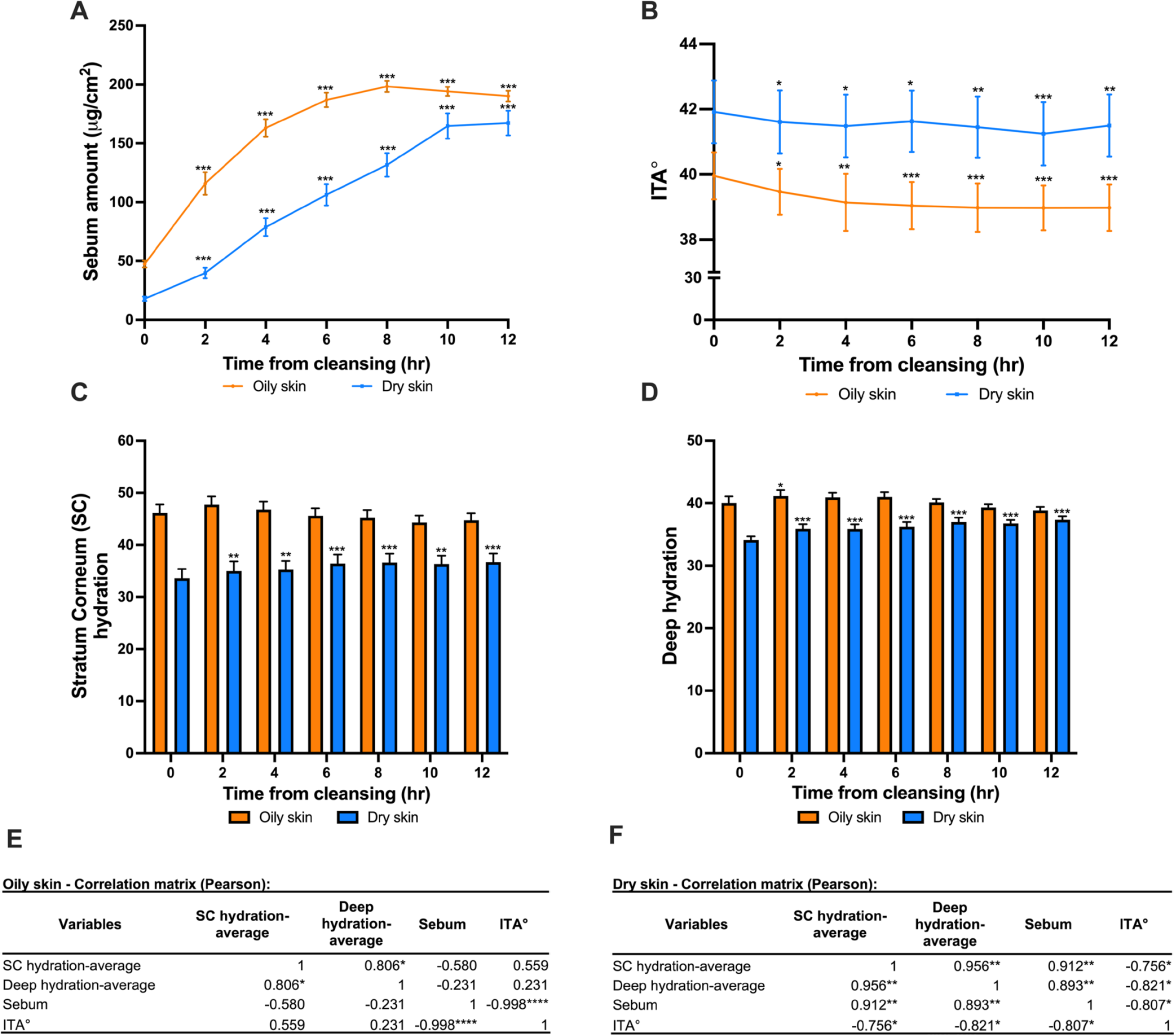
Sebum, skin tone, and hydration over time in both oily and dry skin groups. (A) Sebum measured over 12 h after facial cleansing in the oily and dry skin groups as compared to T0. (B) The ITA° measured over 12 h after facial cleaning in the oily and dry skin groups as compared to T0. (C) The stratum corneum (SC) hydration measured over 12 h after facial cleaning in the oily and dry skin groups as compared to T0. (D) The deep hydration measured over 12 h after facial cleaning in the oily and dry skin groups as compared to T0. (E) The ITA° in relation to the potential influencing parameters for the oily skin group, as determined by the Pearson correlation analysis. (F) The ITA° in relation to the potential influencing parameters for the dry skin group as determined by the Pearson correlation analysis. * denotes statistical significance * < 0.05, ** < 0.01, *** < 0.001, and **** < 0.0001. Individual typology angle (ITA°) * denotes statistical significance, * < 0.05, ** < 0.01, *** < 0.001, and **** < 0.0001.

In the oily skin group, the sebum secretion increased significantly, plateauing after approximately 8 h, which was approximately four times higher than at T0 (Figure 2A). For the dry skin group, the sebum secretion increased significantly, but a plateau was not reached within the 12‐h period. However, the growth rate decreased after T10. At every timepoint analyzed, the amount of facial sebum was higher in the oily skin group than the dry skin group (Figure 2A). In parallel to sebum amount, ITA° decreased dramatically in the first 4 h for the oily skin group (Figure 2B). There was a decrease in skin tone in the dry skin group, which was primarily observed in the first 2 h (Figure 2B). SC hydration did not significantly change from T0 during the 12 h post cleansing in the oily skin group (Figure 2C). However, in the dry skin group, SC hydration significantly increased from 2 h post cleansing (Figure 2C). Deep hydration was only significantly different from T0 at 2 h post‐facial cleansing in the oily skin group whereas in the dry skin group, deep hydration significantly increased from T0 across the 12 h (Figure 2D). Pearson correlation analysis was performed for the oily and dry skin. In the oily skin group, sebum amount showed a strong correlation with ITA° (Figure 2E). In the dry skin group, SC hydration, deep hydration, and sebum amount were strongly correlated with ITA° score (Figure 2F). A subgroup analysis was performed to examine sebum amount, SC hydration, deep hydration, and skin tone (ITA°) across different pore sizes in individuals with oily skin. There was no significant difference in sebum amount, SC hydration, deep hydration, and ITA° value between the pore size subgroups in oily skin, except for a higher sebum value found in the larger pore size group at T4 (Suppl. Figure 3). The correlation results for the different pore size groups confirmed that sebum is the primary factor affecting skin tone in oily skin regardless of pore size (Suppl. Figure 3), while SC hydration and deep hydration are less relevant.

### 4.2 Phase 2 Study

All the respondents (*n* = 200) in Phase 2 self‐reported yellowness as a facial concern. Additionally, over 50% of respondents also reported their own skin concerns with dullness, dark circles, uneven skin tone, enlarged pores, T‐zone blackheads, acne scars, and lackluster skin (Suppl. Figure 2).

#### 4.2.1 The Influence of Pore Size on the Perception of Skin Yellowness

**FIGURE 3:**
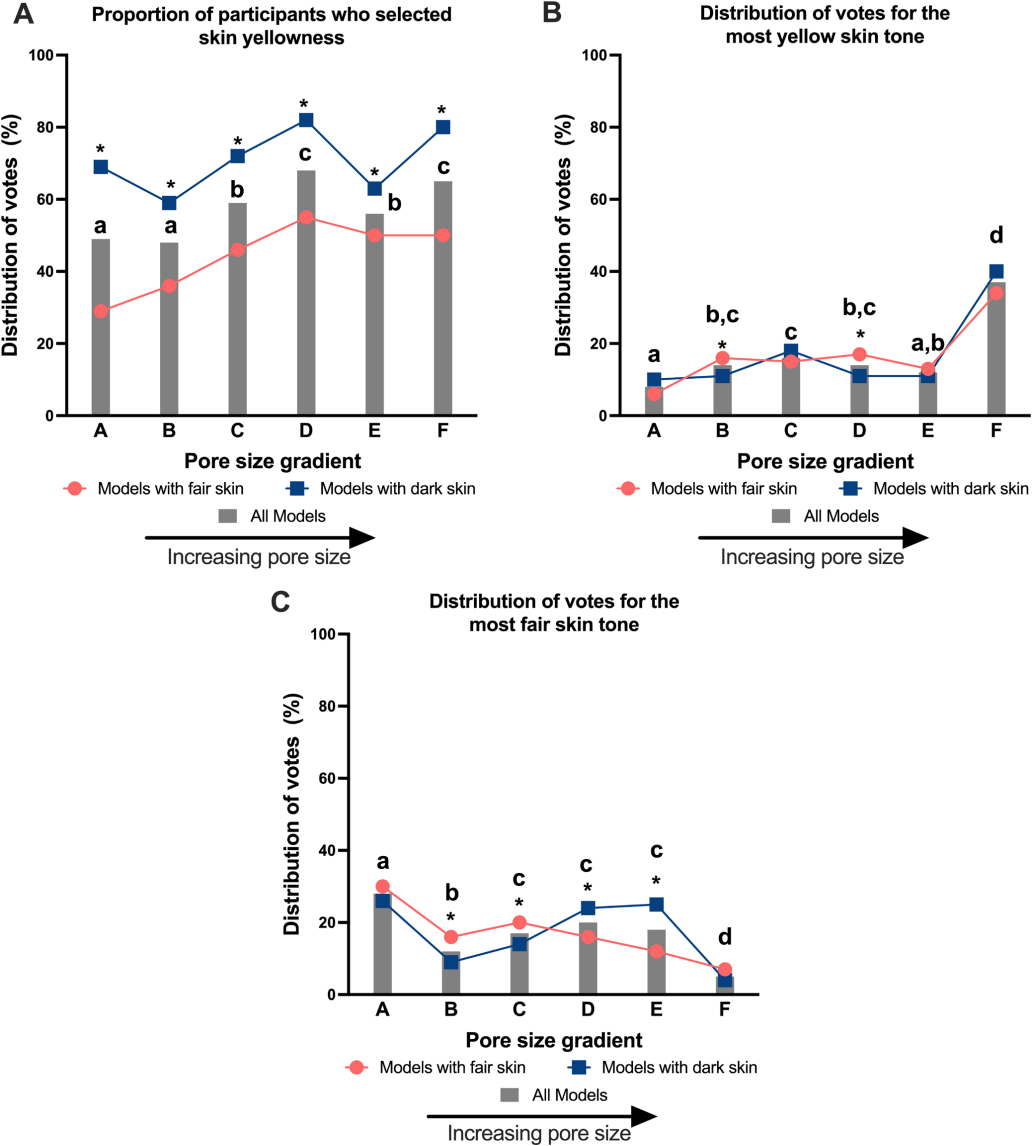
Effect of pore size on perceived skin yellowness. (A) The proportion of participants who perceived skin yellowness across six pore size gradients, presented for all models (bars) and stratified by models with fair skin versus dark skin (lines). (B) The distribution of votes for the “most yellow skin tone” across the six pore size gradient image categories, presented for all models (bars) and stratified by models with fair skin versus dark skin (lines). (C) The distribution of votes for the “fairest skin tone” across the six pore size gradient image categories, presented for all models (bars) and stratified by models with fair skin versus dark skin (lines). Bars not sharing a letter above are significantly different from each other showing differences between pore size gradients. * refers to the line graphs and shows a significant difference in pore size in models with dark skin compared to models with light skin within that gradient. Statistics determined by Cochran's Q test.

Overall, increasing pore size was more often associated with skin yellowness perception. Models with larger pores were perceived to have skin yellowness by more participants than those with smaller pores (Figure 3A). Models exhibiting the two smallest pore size gradients (gradients A and B) had significantly fewer participants perceive them with skin yellowness than those exhibiting larger pore size gradients, particularly gradients D to F (Figure 3A). When asked to select the image with the most yellow skin tone, more than one‐third of participants (37%) chose the image with the largest pore size (gradient F) (Figure 3B), which is significantly higher than the first 5 gradients (gradients A to E). When asked to select the image with the fairest skin tone, 28% of participants chose the image with the smallest pore size (gradient A) (Figure 3C), which is also significantly superior to the other gradients (gradients B to F). Similar trends were seen when results were stratified by skin tone (i.e., models with fair versus dark skin tone) (Figure 3A – C). Significantly more participants perceived yellowness in models with dark skin tone than in models with fair skin tone (Figure 3A).

#### 4.2.2 The Influence of Sebum on the Perception of Skin Yellowness

**FIGURE 4:**
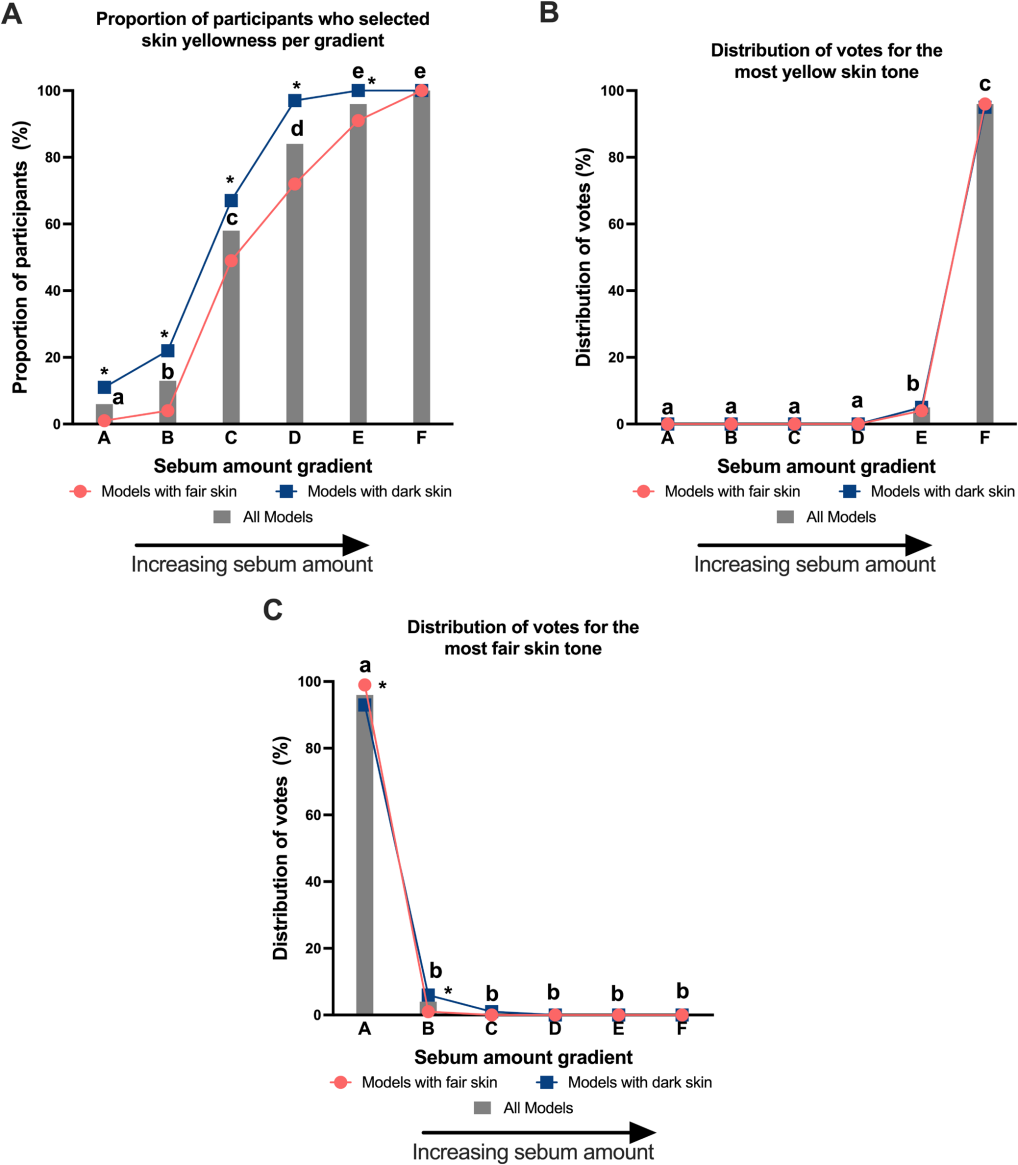
Effect of increasing sebum amount on perceived skin yellowness. (A) The proportion of participants who perceived skin yellowness across six sebum amount gradients, presented for all models (bars) and stratified by models with fair skin versus dark skin (lines). (B) The distribution of votes for the “most yellow skin tone” across the six sebum amount gradient image categories, presented for all models (bars) and stratified by models with fair skin versus dark skin (lines). (C) The distribution of votes for the “fairest skin tone” across the six sebum amount gradient image categories, presented for all models (bars) and stratified by models with fair skin versus dark skin (lines). Bars not sharing a letter above are significantly different from each other showing differences between sebum amount gradients. * refers to the line graphs and shows a significant difference in sebum amount in models with dark skin compared to models with light skin within that gradient. Statistics determined by Cochran's Q test.

Overall, increasing facial sebum amount was more often associated with skin yellowness perception. The number of participants who perceived skin yellowness increased as the amount of sebum increased (Figure 4A). Models in the two lowest sebum amount gradients (gradients A and B) had significantly fewer participants perceive them with skin yellowness than those in the higher sebum amount gradients (Figure 4A). When asked to select the image with the most yellow skin tone, almost all participants (96%) chose the images with the highest sebum amount (gradient F) (Figure 4B). When asked to select the image with the fairest skin tone, almost all participants (96%) chose the images with the lowest sebum amount (gradient A) (Figure 4C). Similar trends were seen when results were stratified by skin tone (i.e., models with fair versus dark skin tone) (Figure 4A – C). Significantly more participants perceived yellowness in models with dark skin tone than in models with fair skin tone (Figure 4A).

#### 4.2.3 The Influence of Pore Size + Sebum on the Perception of Skin Yellowness

**FIGURE 5:**
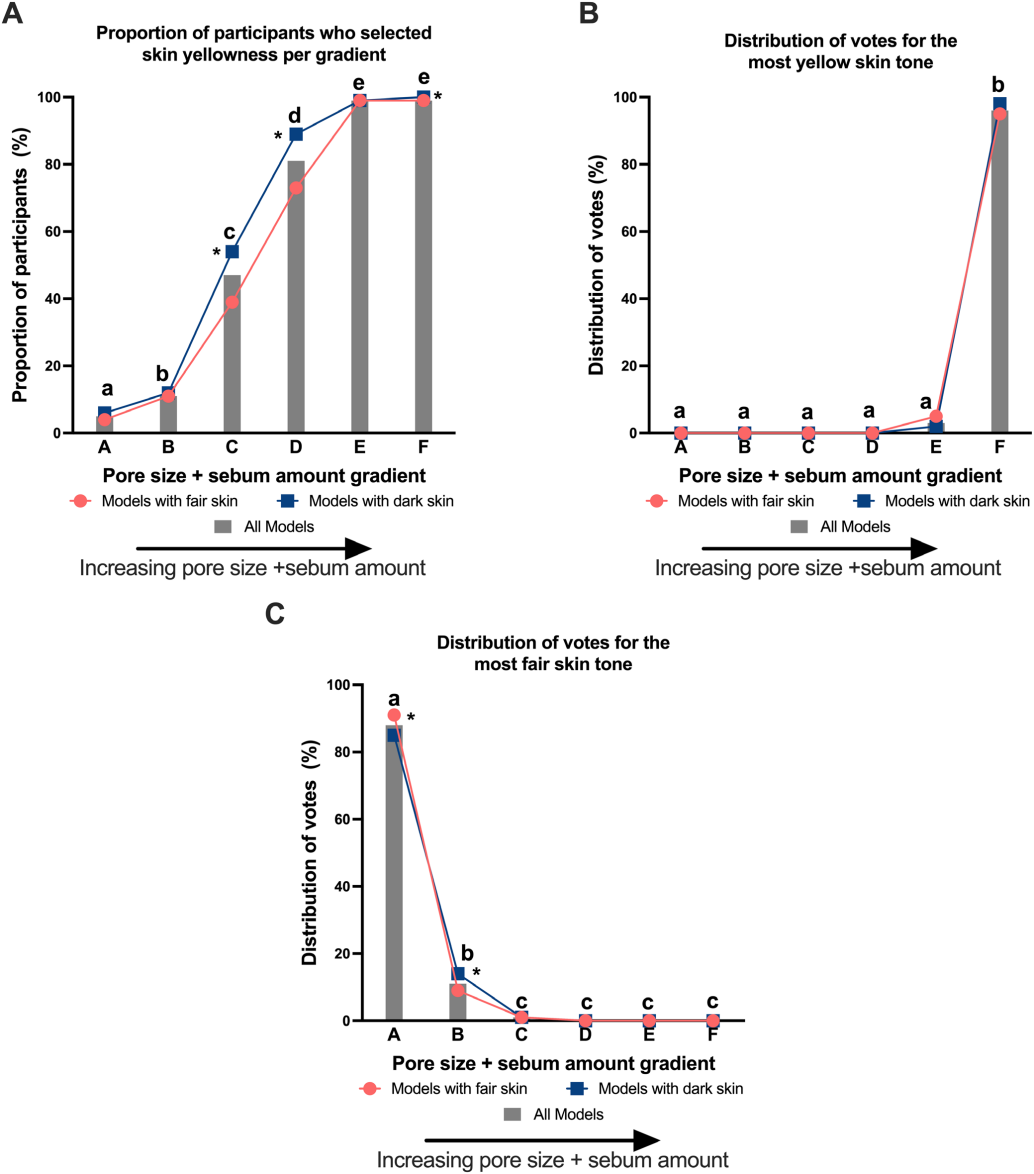
Effect of increasing pore size + sebum amount on perceived skin yellowness. (A) The proportion of participants who perceived skin yellowness across six pore size + sebum amount gradients, presented for all models (bars) and stratified by models with fair skin versus dark skin (lines). (B) The distribution of votes for the “most yellow skin tone” across the six pore size + sebum amount gradient image categories, presented for all models (bars) and stratified by models with fair skin versus dark skin (lines). (C) The distribution of votes for the “fairest skin tone” across the six pore size + sebum amount gradient image categories, presented for all models (bars) and stratified by models with fair skin versus dark skin (lines). Bars not sharing a letter above are significantly different from each other showing differences between pore size + sebum amount gradients. * refers to the line graphs and shows a significant difference in pore size + sebum amount in models with dark skin compared to models with light skin within that gradient. Statistics determined by Cochran's Q test.

In the AI‐generated images for pore size + sebum, both the pore size and the sebum amount increased as the gradient increased. Overall, increasing combined pore size + sebum amount was more often associated with skin yellowness perception. The number of participants who perceived skin yellowness increased as the pore size + sebum increased (Figure 5A). Models in the two smallest pore size + sebum amount gradients (gradients A and B) had significantly fewer participants who perceived them with skin yellowness than those in the higher pore size + sebum amount gradients (Figure 5A). When asked to select the image with the most yellow skin tone, almost all participants (96%) chose the images with the highest pore size + sebum amount (gradient F) (Figure 5B). When asked to select the image with the fairest skin tone, most participants (88%) chose the images with the smallest pore size + sebum amount (gradient A) and 11% chose gradient B (Figure 5C). Similar trends were seen when results were stratified by skin tone (i.e., models with fair versus dark skin tone) (Figure 5A–C). Significantly more participants perceived yellowness in the third and fourth gradients in models with dark skin tone than in models with fair skin tone (Figure 5A).

#### 4.2.4 Comparison of Skin Yellowness Perception Between Skin Parameters

**FIGURE 6:**
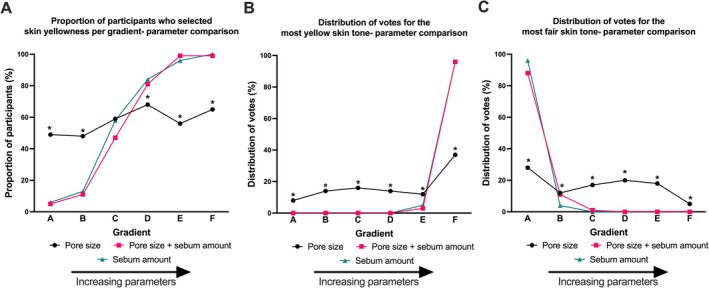
Comparison between parameters on perceived skin yellowness. (A) skin yellowness perception increases with the increasing parameter gradient for sebum amount and pore size + sebum amount, while yellowness perception remains steady across the pore size gradients. (B) gradient F was selected by the majority of participants for most yellowness by both sebum amount and pore size + sebum amount; this pattern was significantly less striking for pore size only. (C) gradient A was chosen as most fair by most participants for both sebum amount and pore size + sebum amount parameters. The selection of fairest skin with increasing pore size was more evenly spread across the gradients. Gradients A to F representing increasing pore size gradients. (*) Pore size was significantly different to sebum amount and pore size + sebum within that gradient, as determined by Cochran's Q test.

Sebum amount and the combination of pore size + sebum amount showed similar influencing trends on participants' perceptions of skin yellowness in the multiple‐choice test (Figure 6A). Consumers were less sensitive to the gradient change in pore size compared to the other two parameters, with a flatter trend observed (Figure 6A). The results of selecting the most yellow and the fairest photographs also showed consistent trends to the multiple‐choice test (Figure 6B,C). However, the effect of pore size was less obvious than that of the other two parameters for both the selection of the most yellow (Figure 6B) and the fairest (Figure 6C).

## 5 Discussion

Skin yellowness is a common cosmetic facial skin concern among Chinese consumers, with skin yellowness increasing with age. Although there are existing cosmetic solutions that focus on whitening and anti‐aging, the underlying causes of skin yellowness may differ for young Chinese consumers, who often face specific skin issues such as excessive sebum secretion and enlarged pores [4, 5, 17]. A better understanding of the underlying factors influencing the perception of skin yellowness in this specific group will help the development of products that resolve or improve consumer skin tone concerns. This study found that increasing sebum and, to a lesser extent, increasing pore size, increased the perception of skin yellowness among young Chinese consumers. This effect was further amplified in consumers with a darker skin tone.

The first phase of the study found a significant difference in the rate of sebum secretion and the pattern of ITA° values (i.e., skin yellowness) between dry and oily skin over the 12 h post‐facial cleansing. In people with oily skin, skin pigmentation increased (as indicated by a decrease in ITA°) predominantly within the first 4 h post‐cleansing. Correspondingly, facial sebum levels had significantly risen within the first 4 h. These findings, supported by a statistically significant correlation (*p* < 0.0001), demonstrated a positive relationship between skin oiliness and skin yellowness. This finding supports previous reports demonstrating that oily skin is associated with increases in sebum secretion [7] and high sebum amount is associated with skin dullness and due to sebum oxidation resulting in a deeper skin color [7]. While the driving factor (sebum production) influencing skin yellowness was clear in the oily group, the relationship was more complicated in the dry skin group. Based on the Pearson correlation results in this study, multiple factors (including sebum and hydration) were found to be associated with skin yellowness (ITA°) in people with dry skin. These findings suggest that the primary factors driving skin yellowness may differ between oily and dry skin. In support of these findings, a previous study found significant differences in pore size, sebum amount, skin roughness, and skin redness between oily and dry skin after a 4‐h period [16]. The findings from this study add to the growing body of knowledge regarding the differences between oily and dry skin. The results from Phase 1 reinforced our understanding of the impact of sebum on skin tone in individuals with oily skin in the short term. Despite there being no clear correlation between pore size and skin tone from Phase 1, pore size is closely linked to sebum production, and the presence of enlarged pores reportedly exacerbates skin yellowness [2, 4, 12, 14, 15, 16, 17]. Therefore, Phase 2 of our study focused on sebum, pore size, and the combination of sebum + pore size. The other parameters (SC hydration and deep hydration) were not chosen for Phase 2 as they demonstrated minimal association with skin tone in the oily skin group.

During the second phase of the study, the participants’ perceptions of the degree of facial skin yellowness increased with increasing severity of the three different factors tested (pore size, sebum amount, and the combination of pore size + sebum amount) on facial skin yellowness. This demonstrated that young Chinese consumers hold a certain degree of discrimination and judgment ability regarding the influencing degrees of all three different factors. Although this effect was not limited by skin tone, consumers with darker skin tones were considered more yellow by respondents. These findings are consistent with those of our previous research, which showed that consumers’ own perceptions of their skin tone and presence of skin yellowness closely aligned with the clinical parameters and measurements of skin yellowness [3]. This is meaningful as the term ‘yellowness’ could be seen as subjective [3], suggesting that consumer perception on skin yellowness is quite complex, informed, and influenced by various skin parameters.

This study found a clear influence of sebum amount and pore size + sebum amount on the perception of skin yellowness by consumers. The proportion of consumers who perceived skin yellowness increased with increasing severity of these factors. Comparatively, both sebum amount and the combination of pore size + sebum amount showed highly consistent patterns in influencing perceived skin yellowness. This indicates that sebum has a dominant effect on consumer perception of skin yellowness when both sebum and enlarged pores are present, while the effect of pore size is less noticeable and overshadowed by sebum. These results closely align with those of previous research showing that sebum oxidation readily causes skin dullness (yellowness) over a short period [7, 8]. Meanwhile, pore size alone was also found to influence skin yellowness perception, albeit to a lesser degree than sebum in the short term since the influence of pore size on the perception of skin yellowness is primarily evident at the high end of the pore gradient. This finding supports those of previous research showing that skin features related to skin reflection influence skin tone perception [17, 24]. Smoother skin is perceived as less yellow, highlighting the importance of minimizing pores, given that pores are a key factor affecting skin texture [17]. This indicates that pore size could result in skin yellowness perception if the enlarged pores continue to worsen without being appropriately addressed.

The findings from this study suggest that skin yellowness perception is triggered not only by aging or a dull skin complexion, but it also occurs with the influence of sebum secretion and enlarged pores for young Chinese consumers. These findings indicate that young Chinese consumers need to take action for oil control and for the prevention of enlarged pore sizes, which could lead to skin yellowness perception. The findings of this study can also be used to develop targeted facial skin care products for young Chinese consumers to combat skin yellowness, aside from common solutions such as whitening and anti‐aging.

## 6 Limitations

Limitations of this study include the use of AI to generate gradient images of model real life skin concerns. Additionally, while this study found differences between oily and dry skin in the primary factors driving skin yellowness perception, there was insufficient data on dry skin to explore and validate its impact on the perception of skin yellowness. Future research could be conducted to gain a deeper understanding of skin parameters associated with perceptual skin yellowness in individuals with dry skin.

## 7 Conclusions

This study found that facial sebum and, to a lesser extent, pore size increase perceived skin yellowness among young Chinese consumers, suggesting that excessive sebum production and pore size could be areas of interest for the development of skin products that target skin yellowness. Young consumers may be able to reduce the perception of skin yellowness primarily by reducing sebum and managing the appearance of enlarged pores. Overall, the results from this study build on the growing knowledge of perceived skin yellowness in young Chinese consumers and will help the cosmetics industry develop better remedies for this skin concern in this age group.

## Author Contributions

S.S., L.R., and B.L.L. performed the research, designed the study, analyzed the data, and edited and approved the manuscript.

## Funding

This work was supported by Groupe Clarins, Singapore.

## Ethics Statement

All participants provided their informed consent before participating in the study. The study protocol was approved by How‐To Science & Technology (Shanghai) Co. Ltd. Ethics Committee (IEC approval number: HT/2024–012).

## Conflicts of Interest

L.R., B.L.L., and S.S. were employees of Groupe Clarins, Singapore, at the time of the study.

## Supporting information


**Data S1:** Supporting Information.

## Data Availability

The data that support the findings of this study are available from the corresponding author upon reasonable request.
