# Supplementary material for: The Impact of Sebum and Pore Size on Consumer Perceptions of Skin Yellowness Among Young Chinese Consumers
Source: J Cosmet Dermatol. 2026 Feb 2;25(2):e70709. doi: 10.1111/jocd.70709 (PMC12865136; doi:10.1111/jocd.70709)
Supplement: Supplementary file 1 — Data S1: Supporting Information. [file JOCD-25-e70709-s001.docx]

**Supplementary Figure 1**. **Models selected from Phase1 for AI-generated image development in Phase 2.**

**Supplementary Figure 2. Participant respondent profile and skin concerns.**

**Supplementary Figure 3. Subgroup analysis by pore size**

**A**

**B**

**C**
